# Personal

**Published:** 1907-05

**Authors:** 


### PERSONAL.

DR. WILLIAM A. EVANS, of Chicago, has been appointed health commissioner of that city by Mayor Busse, who defeated Judge Dunne at the last election. Dr. Evans is one of the most prominent men in his profession in Chicago and, like Dr. Wende, health commissioner of Buffalo, will conduct a sanitary campaign on scientific principles and on energetic fearless lines.

DR. CHARLES A. ELLIS, of Sherman, N. Y., has sold his real estate and transferred his business interests to Dr. James L. Watt, of New York. Dr. Ellis will take a much needed rest after years of professional activity.

DR. ALMON H. COOKE, of Buffalo, announces his removal May 1, 1907, from 410 to 411 Ashland Avenue. Hours: until 9:30 a. m.; 1 to 3 and 7 to 8 p. m.

DR. E. S. MCKEE of Cincinnati, on account of his recent medico-legal writings has been made a member of the Medico-Legal Society of New York and one of the editors of the New York Medico-Legal Journal.

